# The longitudinal effects of education on depression: Finding from the Indonesian national survey

**DOI:** 10.3389/fpubh.2022.1017995

**Published:** 2022-10-20

**Authors:** Bhina Patria

**Affiliations:** Faculty of Psychology, Universitas Gadjah Mada, Yogyakarta, Indonesia

**Keywords:** education, depression, longitudinal study, cross-lagged panel model, longitudinal effects of education on depression

## Abstract

A thorough and continuous investigation of the association between education and depression in Southeast Asia is critical, particularly in Indonesia, where depression is highly prevalent. Despite this, studies on education and depression mainly use a cross-sectional design alone, which cannot control the bidirectionality of the relationship. Therefore, this study investigated the longitudinal effects of education on depression symptoms, based on nationally representative survey data. This study used as its basis a longitudinal socioeconomic and health survey in Indonesia, the Indonesia Family Life Survey (IFLS). The survey collected data through face-to-face interviews with individual respondents and their families. The fourth and fifth waves of IFLS datasets were used in the analysis. A total number of 18,374 adult participants were included in the dataset. Depression symptoms were assessed based on a 10-item version of the CES-D (Center for Epidemiologic Studies Depression) Scale. Education level was the highest level of education attained by the participants. A cross-lagged longitudinal model was tested using structural equation modeling (SEM) or analysis of covariance structure. The results showed that the model of education and depression fits the data well. The fit indices of the model, χ^2^ (1, *N* = 18,374) = 21.592, *p* = 0.001, RMSEA = 0.033, CFI =. 0999, fulfilled the requirements for a good fit. Meanwhile, further analysis of the cross-lagged model revealed that education predicted depression and not the other way around. The standardized regression weights showed that higher education attainment reduces the risk of depression later in life. This study asserts that educational attainment has longitudinal effects on depression. Therefore, expanding the policies surrounding educational opportunity may prevent the onset of depression. This is important, especially in the Indonesian context, where the prevalence of depression among adults is higher than the global average. Access to further education deserves continued consideration in research and policy discussions on mental health and educational system development.

## Introduction

Improving the educational sector is one way to improve public health conditions (1). Studies showed significant and consistent associations between formal educational attainment and health outcomes (2–4), particularly that individuals with better education are healthier and live longer (1, 5). One of the reasons is that better education leads to higher health literacy (6, 7).

Health literacy is the capacity to obtain, process, and understand the basic health information and services needed to make appropriate health decisions (8). Higher health literacy can lead to better access to healthcare and information, medication use, and disease prevention (1, 7). As a result, a higher level of education can create better overall awareness of personal health and make healthcare more accessible (9). On the other hand, people with low health literacy are less healthy, less able to deal with chronic diseases, and have less knowledge about health and more difficulty reading and understanding health information (7).

Studies also showed that education is associated with upward mobility in socioeconomic status, leading to better health. Based on 29 years of cohort data, a longitudinal study concluded that a policy of increased educational opportunities might be effective in breaking the intergenerational transmission of low socioeconomic status and poor health (10).

One of the major issues in global mental health is depression, which affects 4.4% of the world's population (11). This means that over 300 million people are estimated to suffer from depression worldwide. The WHO ranks depression as the single leading contributor to global disability—causing 7.5% of all years lived with disability in 2015 (11)—and depression is also the major contributor to suicide deaths, which number close to 800 thousand per year (11).

Formal education attainment is not only associated with physical health, but also mental health (12). A study using the US Survey of Aging, Status, and Sense of Control showed that years of schooling were associated with a 6% decrease in depression symptoms (12). Another study, based on UK national cohorts, showed that women with lower secondary education have up to 10 percentage points lower depression than women with no qualifications (13).

The relationship between education and mental health is generally categorized into two perspectives: social causation and social selection (4). The social causation perspective asserts that education affects health because it increases socioeconomic conditions, leading to fewer stressors, better coping strategies, better solving of health problems, and lessened risk factors for mental health problems (4). The social selection perspective asserts that early life characteristics trigger the association between education and mental health. In other words, pre-existing mental health conditions are the main reason for eventual termination of schooling (primarily due to functional impairments, stigma, and social exclusion), leading to a worse mental health condition (4).

The prevalence of depression among Indonesian adults ranks higher than the world's average. According to the National Basic Health Survey in 2018, the number had reached 6.1% of the adult population, which means that more than 700 thousand Indonesians were affected by depression (14). A study based on a cross-sectional national survey reported an even higher rate of depression in Indonesian adults-−15% with moderate and 6.9% with severe depressive symptoms, or a total of 21.8% (15). A cross-sectional study focused on Indonesian urban communities reported that the prevalence of depression was 15% and higher among women in the young adult group (16). Another study using a longitudinal method reported a 27.86% rate of depression in young adults, concentrated among lower economic groups (17). These statistics are in accordance with the WHO report, which stated that almost one-third of people with depression live in Southeast Asia (11). The rate was 2.4% in China (18), while a study in Malaysian young adults showed a 25% prevalence of moderate depression and 4.4% of severe depression (19). A nationwide study of Nepalese adults reported a similar number (4.2%) (20). A higher rate, 6.7% in the general population, was reported by a study based on the Korean National Health Survey. Meanwhile, a study based on a national epidemiological survey in Vietnam reported a depression prevalence of 2.8% (21).

Population studies on depression in Indonesia and around the globe were usually conducted with a cross-sectional method (15, 22). One of the weaknesses of cross-sectional correlation studies is directionality: i.e., the existence of a relationship in these studies does not always explain the direction of the relationship (23, 24). Even when a relationship is proven between two variables, the researcher cannot determine whether variable X is the cause of Y or vice versa. Only a few population studies on depression in Asian settings used longitudinal methods (17, 25, 26). However, those studies were not using cross-lagged panel analysis, which has the advantage of controlling the directionality of the relationship. The small subset that did use cross-lagged panel analysis to study depression in Asian settings (27, 28) only focused on undergraduate students over four academic years. Those studies also used one-year intervals that were found to be less optimal in detecting causal effects over time (29). Therefore, the crossed-lagged panel analysis in the present study filled the gap by using data from adult participants with various educational levels and with longer time intervals (i.e., seven years).

Considering the aforementioned conditions, this study aimed to investigate the effect of educational attainment on depression with a longitudinal method, in the hopes that this could fill the methodological gap in previous studies. Therefore, the central research question in this study is: What is the longitudinal effect of education attainment on depression level? In accordance with the literature, the proposed hypothesis is: Better education attainment would reduce the risk of depression later in life for Indonesian adults. This study used a cross-lagged panel analysis with structural equation modeling that has several methodological advantages when analyzing longitudinal data (30). Lastly, it aimed to fill the gaps in the literature on depression in developing country settings, and hopefully contribute to guiding public mental health policy in Indonesia.

## Method

The data were drawn from a longitudinal socioeconomic and health survey in Indonesia, the Indonesia Family Life Survey (IFLS), and were based on a sample of households from 13 provinces, representing about 83% of the Indonesian population (31). The survey collected data through face-to-face interviews with individual respondents and their families. Computer-assisted personal interviewing (CAPI) devices recorded the participants' responses, which assured the effectiveness of the interviewing process and the accuracy of the data.

The present study used the fourth (IFLS4) and fifth wave (IFLS5) datasets of the survey. IFLS4 was fielded in late 2007 and early 2008, while IFLS5 was in late 2014. The sampling scheme was a stratified random sampling based on the strata of provinces and urban/rural locations. IFLS had a high re-contact rate, which is ideal for a longitudinal study; the re-contact rate from IFLS4 to IFLS5 was 90.6% (31) The sampling frame of the survey was considered a comprehensive one because the first IFLS sampling frame was based on the 1993 SUSENAS (National Socioeconomic Survey), which was based on the 1990 census (31).

The IFLS surveys were adequately reviewed and approved by IRBs in the United States and in Indonesia at the Universitas Gadjah Mada (UGM). The ethical clearance number from RAND's Human Subjects Protection Committee (RAND's IRB) was s0064-06-01-CR01.

### Sample and participants

Participants were selected based on the following criteria: (1) age 18 and above; (2) completed the depression scale and education level information in IFLS4 and IFLS5. Based on those criteria, 18,374 participants were selected for this study. The participants consisted of 9,798 (53.3%) females and 8,576 (46.7%) males, with an average age of 34.64 (*SD* = 12.95). Table 1 depicts the sociodemographic data of the participants.

**Table 1:** Sociodemographic data.

**Variable**	***N***	**%**
**Sex**		
Male	8,576	46.7
Female	9,798	53.3
Education level (Years of Schooling) in IFLS4 (2007) ^*^	(*M* = 9.25; *SD* = 3.16)	
Elementary school (6 years)	7,372	40.1
Junior high school (9 years)	3,839	20.9
Senior high school (12 years)	5,857	31.9
Bachelor's degree (16 years)	1,263	6.9
Master's degree (18 years)	43	0.2
Education level (Years of Schooling) in IFLS5 (2014)^*^	(*M* = 9.45; *SD* = 3.35)	
Elementary school (6 years)	7,203	39.2
Junior high school (9 years)	3,736	20.3
Senior high school (12 years)	5,591	30.4
Bachelor's degree (16 years)	1,700	9.3
Master's degree (18 years)	140	0.8
Doctoral degree (22 years)	4	0.0
Depression symptoms^*^		
IFLS4 (2007)	(*M* = 13.9; *SD* = 3.5)	
IFLS5 (2014)	(*M* = 16.2; *SD* = 4.8)	
Total	18,374	100.0

* Arithmetic mean and standard deviation.

### Measurement

Depression was measured by a 10-item version of the CES-D (Center for Epidemiologic Studies Depression) Scale (32), which is a modification of the 20-item version. The reliability and validity of the CES-D are well-established in various samples (33, 34). The psychometric properties of the scale have been evaluated and found to be good (35). CES-D in this study was presented in Bahasa Indonesia, and the internal consistency Cronbach's Alpha = 0.757. The depression data in ILFS4 and IFLS5 were labeled as Depression_1_ and Depression_2_, respectively.

Education level was the highest level of education attained by the participants. The data were converted to years of schooling based on the Indonesian education system. For example, 6 years of schooling indicates elementary school graduates while 16 years of schooling indicates a bachelor's degree. Most of the participants were at the primary and secondary education level (93% in the fourth wave and 90% in the fifth wave), and only a few had higher education titles (7% in the fourth wave and 10% in the fifth wave) (see Table 1). The education level data in ILFS4 and IFLS5 were labeled as Education_1_ and Education_2_, respectively.

### Data analyses

The longitudinal effects of education on depression were analyzed using a cross-lagged panel model. This type of model allows a researcher with longitudinal data to determine, across time, if Variable X is a more likely cause of Variable Y or if Y is a more likely cause of X (30). Cross-lagged models have been commonly used in longitudinal research to investigate the direction of influence between two variables (36–38).

In the present study, the cross-lagged panel models were tested using structural equation modeling or analysis of covariance structure. To assess the fitness of a model, it is necessary to report fit statistics such as Chi-square value and degrees of freedom; the CFI (comparative fit index) or TLI (Tucker-Lewis Index); and RMSEA (root mean square error of approximation) (39, 40). For a model with < 12 observed variables and cases of more than 250, the suggested fit index values are CFI ≥ 0.97 and RMSEA < 0.07 (39).

## Results

**Figure 1:**
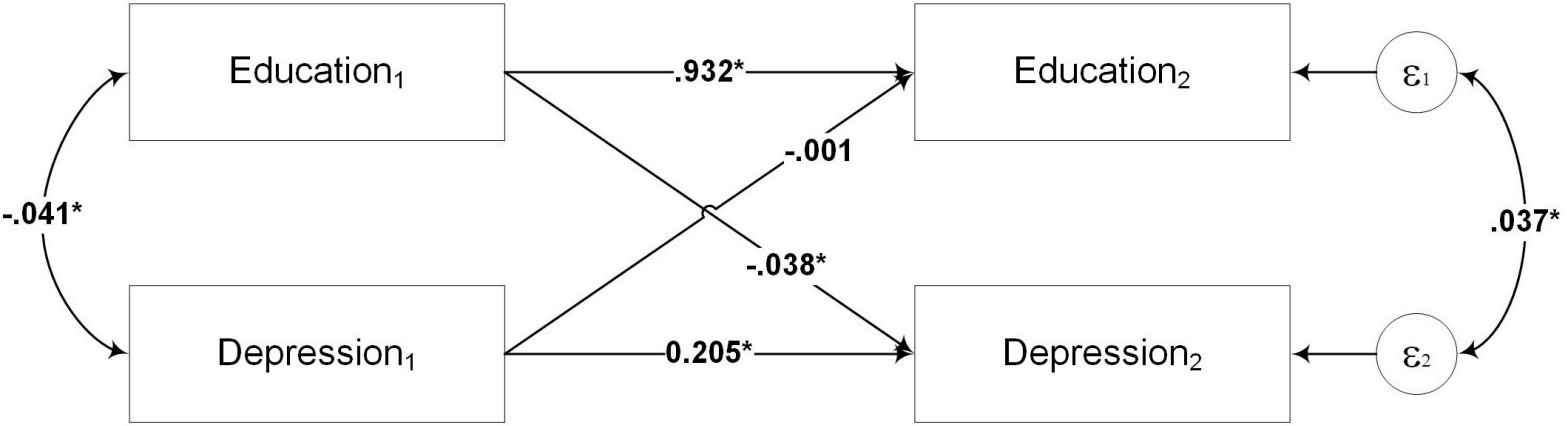
Model 1 the path analytic model of the cross-lagged relationships between education and depression. Education_1_ and Education_2_ refer to education levels at Time 1 and Time 2, respectively. Depression_1_ and Depression_2_ refer to depression at Time 1 and Time 2, respectively. Time 1 is the time of IFLS4 (2007), and Time 2 is the time of IFLS5 (2014). All paths are standardized. ^*^p < 0.01.

This study implemented a two-wave, cross-lagged panel model, in which the variables of education level and depression were each evaluated at two points in time. Time 1 refers to the data from IFLS wave 4 (2007) and Time 2 refers to the data from IFLS5 (2014). The model, graphically displayed in Figure 1, was fitted using Stata 13.1 by the method of maximum likelihood.

Table 2 revealed that, when fitted to the data, Model 1 was identified as a saturated or just-identified model, characterized by zero degrees of freedom and χ^2^ (39). A saturated model is not useful for testing a theory because the circumstance determines the fit (39); it occurs when the number of unique variances or covariances equals the number of estimated parameters (39). Therefore, Model 1 was modified by removing the covariance between the error terms of the variables in Time 2. The results showed that Model 2 fits the data well, since the fit indices, χ^2^ (1, *N* = 18,374) = 25.397, *p* = 0.001, RMSEA = 0.036, CFI = 0.999, fulfill the requirements for a good model fit (39).

**Table 2:** Fit statistics of the education and depression models.

**Model**	***N***	**χ^2^**	**df**	**RMSEA**	**CFI**
Model 1	18,374	0	0	0	1
Model 2	18,374	25.397	1	0.036	0.999

In a cross-lagged panel design, researchers are generally interested in examining the direct paths between the variables. There are two path types: those within each variable and those between variables. The within variable, or autoregressive paths, are the paths that link the same variable measured at two different times (e.g., the path between Education_1_ and Education_2_). These paths provide information about the relative stability of the construct, with higher values indicating better stability (30). The paths measured across variables (e.g., between Education_1_ and Depression_2_) are the essence of the cross-lagged analysis. They provide information about the degree to which one variable is a stronger temporal predictor: does education predict depression or vice versa?

As shown in Table 3, the standardized regression values are significant for the path from Education_1_ to Depression_2_ (β = −0.038 *p* = 0.001), whereas the path from Depression_1_ to Education_2_ is not significant (β = −0.001 *p* = 0.595). These results suggest that depression at Time 2 is far better predicted from the level of education at Time 1 than education at Time 2 is predicted from depression at Time 1. The standardized regression weight showed a negative value, meaning that higher education attainment reduces the risk of depression later in life.

**Table 3:** Standardized regression weight of the effects of education on depression of Model 1 and Model 2^*^.

	**β**	**SE**	***p***
Education_1_ → Depression_2_	−0.038	0.072	0.001*
Depression_1_ → Depression_2_	0.205	0.007	0.001*
Education_1_ → Education_2_	0.932	0.001	0.001*
Depression_1_ → Education_2_	−0.001	0.002	0.595
Depression_1_↔Education_1_	−0.041	0.007	0.001*

All paths in Model 1 and Model 2 have the same regression weight.

* Significant at the 0.01 level.

## Discussion

Previous studies on education and depression in Indonesia were mainly conducted with a cross-sectional method, which failed to distinguish the directionality of the relationship. This study aimed to investigate the longitudinal effects of educational attainment on depression based on Indonesia's national survey data. Using a cross-lagged panel analysis with structural equation modeling (SEM), this study strived for a better method of analyzing the relationship between education and depression. The results showed that the cross-lagged theoretical model of education attainment's effects on depression fits the data well. Education attainment predicts depression and not the other way around.

One of the strengths of this study is the use of structural equation modeling (SEM) to analyze the cross-lagged panel model. SEM was developed to allow researchers to establish causal relationships between variables (39). SEM models are often referred to as causal models because they generally postulate that one or more independent variables are a cause of one or more dependent variables (30). However, the causal inference is not the same as the conventional one involving a controlled condition in an experimental study. In an experimental study, causal inference is characterized by (1) manipulating the independent variable and observing the dependent variable afterward; (2) the variation in the dependent variable being related to the variation in the dependent variable; and (3) reducing the plausibility of other explanations for the effects (41). SEM models are usually used in non-experimental conditions, thus limiting the causal inference power. However, SEM can treat a relationship between variables as causal based on four types of evidence: covariation, sequence, non-spurious covariation, and theoretical support (39). Therefore, it is important to state that the use of SEM in this study can only establish a few conditions necessary for determining causality, i.e., resolving the issues of temporal precedence.

The results of this study corroborate the findings of previous work on the relationship between education and depression. Studies have shown that the association of education with mental health is strong and constant over time (2–4). Studies of education's effects on depression have usually been part of a longitudinal population study investigating the effects of socioeconomic status on mental illness. A longitudinal study based on an acute psychiatric hospitalization database revealed a strong and consistent negative correlation between socioeconomic conditions and mental illness (42). Socioeconomic status impacts the development of mental illness both directly and indirectly through the adverse, economically stressful conditions among lower-income groups (42). The findings supported the role of social causation in the association between education and mental illness.

In accordance with the present results, a longitudinal study in the Indonesian setting demonstrated that education positively affects mental health (25). However, the study focused on the effects of parental education attainment on their children's depressive symptoms. The study used a structural equation model to examine the hypotheses but not a cross-lagged panel model. One of the advantages of using a cross-lagged panel model is the ability to capture between-person differences (or inter-individual differences) and within-person fluctuation over time; this gives a better estimation of the predictors' effects on the outcome (29). Few previous longitudinal studies on depression in Asian settings used cross-lagged panel analysis on mental health. One such study investigated the relationship between self-esteem and depression (27), and another investigated the relationship between self-esteem and anxiety (28). Those studies used the same data set from undergraduate students tracked annually over four academic years. Compared to those studies, the present study proposed a better technique using a more comprehensive set of participant education levels, i.e., from elementary school up to a doctoral degree. The present study also used a longer time interval (i.e., 7 years) in the cross-lagged panel model, which is considered more optimal in detecting causal effects over time (29). Furthermore, the present study used face-to-face interviews, thus providing more valid and reliable data compared to the self-reported scales used in the previous studies.

Although studies on the effects of education on depression are well-documented, the causal mechanism is still unsettled. From the socioeconomic perspective, researchers argue that education affects health because more education boosts social and economic means, leading to fewer stressors, better coping strategies, and more autonomous lifestyles (4). From the psychological and interpersonal perspective, individuals with higher education are seen as having better coping resources, problem-solving and cognitive abilities to prevent adverse health consequences (9). Furthermore, educated individuals tend to have healthier behaviors (9). Another longitudinal study on the socioeconomic pathways to depression revealed that the effect of education on depressive symptoms was mediated by income (10). Parents' education also affected participants' educational attainment, affecting income and finally depressive symptoms (10).

The findings of this study should be evaluated with some limitations in mind. In this study, the effect of other variables (e.g., income, health, lifestyles) on the relationship between education and depression was not controlled. This is mainly due to the limitations of cross-lagged panel analysis, which “in theory, assumes all possible variables were measured and included in the model” (43). Another limitation of this study is the measure of depression which was based on a self-rating scale. Even though the CES-D Scale is a validated instrument, its purpose is limited to depression screening. The ideal condition for depression diagnosis is through the assistance of a mental health professional. However, in a population study, this would be difficult to conduct considering the enormous number of participants. Additionally, the data collection process in IFLS used face-to-face interviews with household members. This means that participant interviews may have been conducted in the presence of other family members, and thus responses could be affected by social desirability—i.e., the participants could have presented themselves in a generally favorable rather than a fully truthful fashion (44).

## Conclusion

This study asserts that educational attainment has longitudinal effects on depression. Thus, higher levels of education will reduce the risk of depression in later life, and expanding policy related to educational opportunity might therefore aid in preventing its onset. This is important especially in the Indonesian context, where the prevalence of depression among adults is higher than the world's average. This study lends support to the relationship between educational attainment and depression. Access to further education therefore deserves continued focus in research and policy discussion on mental health and educational system development.

The findings from this study make several contributions to the current literature. First, it uses a nationally representative longitudinal adult data. This is important because most studies on depression used the cross-sectional method. Second, this study used face-to-face interviews, thus providing more valid and reliable data. Third, the use of cross-lagged panel analysis with structural equation modeling better estimates the effects of education on depression and overcomes the limitations of previous study methods, e.g., by resolving the issues of temporal precedence in causal inference.

## Data availability statement

Publicly available datasets were analyzed in this study. This data can be found here: https://www.rand.org/well-being/social-and-behavioral-policy/data/FLS/IFLS/access.html.

## Ethics statement

The studies involving human participants were reviewed and approved by RAND's Human Subjects Protection Committee (RAND's IRB) was s0064-06-01-CR01. The patients/participants provided their written informed consent to participate in this study.

## Author contributions

The author confirms being the sole contributor of this work and has approved it for publication.

## Funding

This study was funded by the Faculty of Psychology, Universitas Gadjah Mada, Yogyakarta, Indonesia. The publication process was supported by the Directorate of Research, Universitas Gadjah Mada, Yogyakarta, Indonesia. The funding body had no involvement in the study design, data collection, analysis, or manuscript preparation.

## Conflict of interest

The author declares that the research was conducted in the absence of any commercial or financial relationships that could be construed as a potential conflict of interest.

## Publisher's note

All claims expressed in this article are solely those of the authors and do not necessarily represent those of their affiliated organizations, or those of the publisher, the editors and the reviewers. Any product that may be evaluated in this article, or claim that may be made by its manufacturer, is not guaranteed or endorsed by the publisher.
